# Association between androgenetic alopecia and psychological well-being: a systematic review and meta-analysis

**DOI:** 10.3389/fpsyt.2025.1705957

**Published:** 2025-11-26

**Authors:** Yuan Kong, Yutong Shang, Liuwei Zhang

**Affiliations:** 1 Department of Dermatology, Beijing Changping Hospital of Integrated Chinese and Western Medicine, Beijing, China; 2 Key Laboratory of Exercise and Physical Fitness, Beijing Sport University, Ministry of Education, Beijing, China; 3 Department of Physical Fitness and Health, School of Sport Science, Beijing Sport University, Beijing, China

**Keywords:** androgenetic alopecia, psychology, anxiety, depression, stress, meta

## Abstract

**Background:**

Androgenetic alopecia (AGA) is a prevalent chronic condition with health burden, yet its association with psychological well-being remains inconsistent. This study systematically examines differences in psychological well-being between AGA patients and non-AGA controls, focusing on symptoms of anxiety, symptoms of depression, stress, and other psychological issues.

**Methods:**

Following the Preferred Reporting Items for Systematic Reviews and Meta-Analyses guidelines, a systematic search was conducted across 7 databases (CNKI, Wanfang, PubMed, Web of Science, Cochrane, EMBASE, EBSCO series) from their inception to June 30, 2025. A total of 13 studies were included, comprising 2,737 AGA patients and 17,382 controls. Study quality was assessed using the Agency for Healthcare Research and Quality (AHRQ) tool for cross-sectional studies and Newcastle-Ottawa Scale (NOS) for case-control studies. Effect sizes were pooled using a random-effects model, supplemented by sensitivity analysis and publication bias assessment to ensure the robustness of the results.

**Results:**

Compared to non-AGA controls, AGA patients demonstrated significantly higher levels of generalized anxiety symptoms (pooled SMD = -0.50, 95% CI [-0.99, 0.00], p = 0.05), social anxiety symptoms (pooled SMD = -0.50, 95% CI [-0.84,-0.16], p = 0.004), depression symptoms (pooled SMD = -0.38, 95% CI [-0.65,-0.12], p = 0.004), and perceived stress (pooled SMD = -1.09, 95% CI [-1.43,-0.74], p < 0.001). However, no significant difference was observed in general distress (pooled SMD = -0.01, 95% CI [-0.39,0.37], p = 0.97). Qualitatively, AGA patients also exhibited reduced self-esteem, life satisfaction, and emotional intelligence dimensions, along with greater body image dissatisfaction, somatization, interpersonal sensitivity, and psychoticism.

**Conclusion:**

AGA patients experience increased levels of symptoms of anxiety, symptoms of depression, stress, body image dissatisfaction, somatization, interpersonal sensitivity, and psychoticism, as well as decreased self-esteem, life satisfaction, emotional intelligence, self-actualization, problem-solving ability, optimism, and happiness. In the future, clinical treatment should focus on patient psychological well-being through assessments and timely interventions, and further research should be conducted to provide precise clinical guidance.

**Systematic Review Registration:**

[https://www.crd.york.ac.uk/PROSPERO/], identifier [CRD420250655604].

## 1 Introduction

Hair, a vital epidermal appendage of the head and face, performs essential physiological functions such as scalp protection, sensory perception, and sebum regulation. Additionally, its biophysical and biochemical properties serve as health indicators for disease screening (1, 2). As a core component of body image, it also influences psychological well-being (3, 4), a multidimensional concept that, in the context of our study, includes dimensions that encompass both the absence of psychological illness or distress and positive states and functions. Among hair disorders, androgenetic alopecia (AGA) is clinically significant due to its high prevalence and substantial health burden. It is a non-scarring hair loss that usually begins in puberty, characterized by gradual hair loss and miniaturization of hair follicles. In men, it shows as a receding frontal hairline and/or thinning hair on the top of the head. In women, it shows as thinning hair on the top of the head, but the hairline usually remains unchanged (5). Epidemiological studies show that the incidence rate of AGA is higher in men than in women at 24.88% and 5.06% respectively (6). Specifically, the prevalence is about 20% in Chinese men and ranges from 22.6% to 65% in African populations. Moreover, the incidence rate increases significantly with age—reaching approximately 50% by age 50 and 80% by age 80 in European men, increasing to 29% to 42% in women aged 70 years and over (5, 7, 8). Pathologically, AGA arises from genetic predisposition, dysregulated androgen metabolism, growth factor imbalances, and follicular microenvironment disruption—a process that drives progressive hair miniaturization while being associated with several systemic diseases such as cardiovascular disease and metabolic syndrome (8). Psychologically, AGA also profoundly impacts psychological health, frequently leading to diminished self-esteem, anxiety, and depression, which significantly compromise quality of life and social functioning (4).

Research on the psychological impact of AGA on patients dates back to the 1990s. Early studies revealed that, compared with non-balding individuals and those with low hair loss, patients with advanced hair loss experienced significantly greater stress related to socioemotional functioning, self-esteem, positive life events, and coping behaviors (4). Subsequent studies have shown that AGA patients may experience psychological problems such as anxiety, helplessness, decreased self-esteem, and even depression due to their dissatisfaction with their appearance and concerns about negative evaluations or social rejection from others (3, 4, 9). For instance, a 2023 study involving 1,230 male patients diagnosed with AGA found that 95% experienced stress and 78% felt embarrassed by their hair loss (10). Lin et al. investigated 530 employees at a medical institution and found that, compared with individuals without hair loss, AGA patients exhibited higher levels of somatization, depression, and hostility (11). Although previous studies have explored the relationship between AGA and various dimensions of psychological well-being, the findings remain a matter of debate. Regarding depression specifically, Noha et al., employing the Beck Depression Inventory, found that women with AGA had higher depression levels than healthy female volunteers of the same age (12). Conversely, He et al., using the Patient Health Questionnaire, observed no significant difference in depression levels between students with AGA and those without AGA among the freshmen of the two universities (13). Sinikumpu et al. also found that hair loss severity was not significantly associated with depression levels among 46-year-old men in the Northern Finland 1966 Birth Cohort, as assessed by the Beck Depression Inventory (14). Additionally, a systematic review and meta-analysis concluded that the link between AGA and depression was not statistically significant (3). These inconsistent results highlight the need for further research.

In addition to the contradictory conclusions drawn from prior research across diverse regions and populations, current research on the psychological well-being of the AGA group still has several limitations, mainly in the following aspects: (a) existing studies on AGA mostly focus on physiology and drug treatments, neglecting psychological well-being factors or only considering them as part of quality of life studies; (b) previous studies often examine a single psychological well-being issue in AGA populations, lacking comprehensive comparisons with non-AGA individuals; (c) past studies have limited sample sizes, and the research results are inconsistent, failing to provide sufficient research evidence; (d) previous systematic reviews lack Chinese data, and focus on a limited range of dimensions. For example, the study by Huang et al. (9) primarily utilized data from Western populations, with insufficient representation from China and other Asian countries, and lacked a non-AGA control group for comparison. The research by Frith et al. (3) focused exclusively on males and conducted quantitative analysis solely on depression and quality of life within the patient population, which limited the scope of their analysis. The review by Aukerman et al. (4) was qualitative in nature and did not include quantitative analysis. Therefore, this study compares the psychological well-being of AGA and non-AGA populations by including a broader range of studies from multiple databases, integrating data from multiple scales, incorporating data from China, and examining both male and female patients, thereby providing a theoretical basis for the prevention and intervention of AGA.

## 2 Materials and methods

This study adhered strictly to the Preferred Reporting Items for Systematic Reviews and Meta-Analyses (PRISMA) guidelines. The registration number of this meta-analysis on PROSPERO is CRD420250655604 (Available from https://www.crd.york.ac.uk/PROSPERO/view/CRD420250655604).

### 2.1 Literature search

The electronic databases CNKI, Wanfang, PubMed, Web of Science, Cochrane, EMBASE, and EBSCO series were searched to find all literature related to AGA and psychological well-being. Search for the following keywords and their extensions using the corresponding search formula: “androgenetic alopecia/seborrheic alopecia (in Chinese)/pattern alopecia/hair loss”, “mental disorders/psychology/psychological/emotion/psyche/quality of life/anxiety/depression/stress/self-concept/self-esteem/risk factors” and “questionnaire/scale/inventory/index”. The search period was from the establishment of the databases to June 30, 2025. The language was limited to Chinese and English. Specific search formulas can be viewed in **Supplementary Material**.

### 2.2 Inclusion and exclusion criteria

Inclusion criteria: Studies were included if they (a) were classified as observational studies (Cross-sectional study, case-control study, cohort study), thereby avoiding the potential influence of interventions on the true association between the variables; (b) involved populations in the AGA group and the non-AGA group diagnosed by professionals; (c) clearly specified the tools for assessing psychological well-being and presented the data from the assessment scales; (d) reported mean and standard deviation results or provided data that could be calculated.

Exclusion criteria: Studies were excluded if they (a) were randomized controlled trial studies; (b) involved subjects with clinically diagnosed mental disorders, such as anxiety disorders and depressive disorders; (c) lacked a non-AGA group or did not report relevant results of the control group population; (d) were not reported in Chinese or English.

In our study, psychological well-being dimensions include: (a) Emotional States: Covers both positive emotions (such as optimism, sense of happiness, and life satisfaction) and negative emotions (such as anxiety, depression, stress, somatization, and obsessive-compulsive symptoms); (b) Self-Awareness and Self-Evaluation: Includes self-esteem, self-actualization, self-regard, and emotional self-awareness; (c) Interpersonal Relationships: Involves good interpersonal relationships, empathy, and social support; (d) Psychological Resilience and Adaptability: Includes the ability to cope with stress, impulse control, reality testing, problem-solving, stress tolerance, and the ability to adapt to environmental changes; (e) Psychological Growth: Involves self-awareness, self-development, and self-actualization; (f) Life Satisfaction: Overall satisfaction with and sense of happiness in life; (g) Psychosocial Functioning: Includes social adaptability and social participation; (h) Cognitive and Behavioral Health: Covers clear thinking, good judgment, focused attention, appropriate behavior, and the absence of significant behavioral problems (such as impulsive behavior and obsessive-compulsive behavior); (i) Overall Psychological Health Assessment: Includes the Global Severity Index (GSI) and assessments using psychological health scales, etc.

Two researchers independently performed a literature search and screening across 7 databases, using a customized search strategy for each. They screened titles and abstracts based on predefined criteria, excluded ineligible materials like conference abstracts, books and patents, and removed duplicates. The researchers then assessed the full texts of potentially relevant studies against inclusion criteria related to methodology, participant characteristics, and study data. Additionally, they reviewed the reference lists of selected articles and relevant reviews to identify further potential studies. In the final phase, a third researcher reviewed the articles simultaneously. In case of any conflicts, the third researcher made the final decision.

### 2.3 Data extraction

Data extraction was extracted by one researcher and confirmed by another. Using Excel, information such as the first author, publication year, country, ethnicity, study type, participants resource, sample size, participants’ gender and age, psychological assessment scales, and the reported mean and standard deviation of the scales were extracted. If a single study contains multiple independent AGA or non-AGA groups, each group will be considered as an independent study for analysis. If relevant data for the meta-analysis were missing from the paper, the authors were contacted to obtain the necessary data.

### 2.4 Quality assessment

The quality of the included studies was assessed using the Agency for Healthcare Research and Quality (AHRQ) tool for cross-sectional studies and the Newcastle-Ottawa Scale (NOS) for case-control or cohort studies. Any discrepancies were resolved by a third author.

The AHRQ consists of 11 items, with options of “Yes”, “No”, or “Unclear”. Items rated as “No” or “Unclear” receive a score of “0,” while items rated as “Yes” receive a score of “1.” The maximum score is 11, categorized into three quality levels: 0-3 (low quality); 4-7 (moderate quality); 8-11 (high quality) (15). The NOS evaluates study quality through three main aspects: Selection, Comparability, and Outcome. The total score is 9, and the score for each aspect can be adjusted according to the specifics of the study. Typically, studies scoring 7–9 are considered high quality, 4–6 as medium quality, and less than 4 as low quality (15). Medium and high quality literature is sufficient to be included in the meta-analysis.

### 2.5 Statistical analysis

When the number of studies in a particular dimension reached 3 or more, we conducted a meta-analysis on that dimension. Ultimately, 5 dimensions were included in the meta-analysis: symptoms of generalized anxiety (10 studies), symptoms of social anxiety (3 studies), symptoms of depression (8 studies), perceived stress (6 studies), and general distress (5 studies). These dimensions were categorized based on the assessment content and characteristics of the respective scales used in the studies (16).

The standardized mean difference (SMD) is used as an effect size to compare the psychological well-being status between AGA group and non-AGA group. The SMD is calculated as the difference in means between the two groups divided by the pooled standard deviation. When the mean and standard deviation (SD) were not directly reported, the SD was derived from the standard error of the mean (SEM) or 95% confidence intervals (CI) using the formulas specified in the Cochrane Handbook for Systematic Reviews of Interventions [ $SD=SEM*\sqrt{n}$ or $SD=\sqrt{n}*(upper\;limit-lower\;limit)/3.92]$(17). Additionally, when scores were presented as medians along with the 25th and 75th percentiles, they were transformed into mean ± SD using the method described by Wan et al. in the Cochrane Handbook (18). Subsequently, the standardized mean difference (d) is converted to Hedges’s g to correct for small sample bias. Following this, the pooled effect is calculated using either a fixed-effect model or a random-effects model, depending on the results of the heterogeneity analysis. Heterogeneity is assessed using I², with a fixed-effect model employed when I² ≤ 50% and a random-effects model when I² > 50%. Additionally, sensitivity analysis is conducted to test the stability of the results, and funnel plots as well as Egger’s and Begg’s tests are used to estimate publication bias, with a significance threshold of p < 0.05. In this process, Review Manager 5.4 is utilized for meta-analysis and heterogeneity assessment, while Stata 17.0 is used for sensitivity analysis and publication bias testing.

## 3 Results

### 3.1 Study characteristics and quality assessment

The initial search yielded 5,124 related articles in the database. After excluding 5,111 articles, a total of 13 articles were finally included, comprising 17,382 non-AGA controls and 2,737 AGA patients. 9 studies were conducted in China (13, 19–26), 2 in Egypt (12, 27), 1 in America (28) and 1 in Canada (29). Each study was confirmed based on the clinical criteria for AGA. Of these, 9 studies were diagnosed by specialist doctors in the dermatology or alopecia clinic (12, 13, 20, 23–25, 27–29), and 4 studies were diagnosed as AGA by the researchers based on the AGA diagnosis guidelines (19, 21, 22, 26).

In the included studies, researchers used various validated psychological well-being questionnaires to assess psychological states over different time ranges, focusing on current psychological conditions. For symptoms of anxiety, the Self-Rating Anxiety Scale (SAS) was used in five studies (20–23, 25), while the Beck Anxiety Inventory (BAI) (12) and the 2-item Generalized Anxiety Disorder scale (GAD-2) (13) were also employed. For symptoms of depression, the Self-Rating Depression Scale (SDS) was used in three studies (21, 22, 25), and other tools included the Beck Depression Inventory (BDI) (12), the PHQ-2 (13), and the Center for Epidemiologic Studies Depression scale (CES-D) (24). For stress, the Perceived Stress Scale (PSS) (12) and the Impact of Event Scale (IES) (28) were used.

In multidimensional assessments, tools like the Depression, Anxiety, and Stress Scales (DASS-21) (19), the Revised Symptom Checklist-90 (SCL-90-R) (26), the Emotional Quotient Inventory (EQ-I) (29), and the World Health Organization Quality of Life Brief version (WHOQOL-BREF) (27) were employed to evaluate comprehensive psychological health. Additionally, Cash TF et al. (28) used the Texas Social Behavior Inventory (TSBI), the Self-Consciousness Scale (SCS), and two multi-item indices to assess individuals’ social behavior, self-awareness, and psychological status.

**Figure 1:**
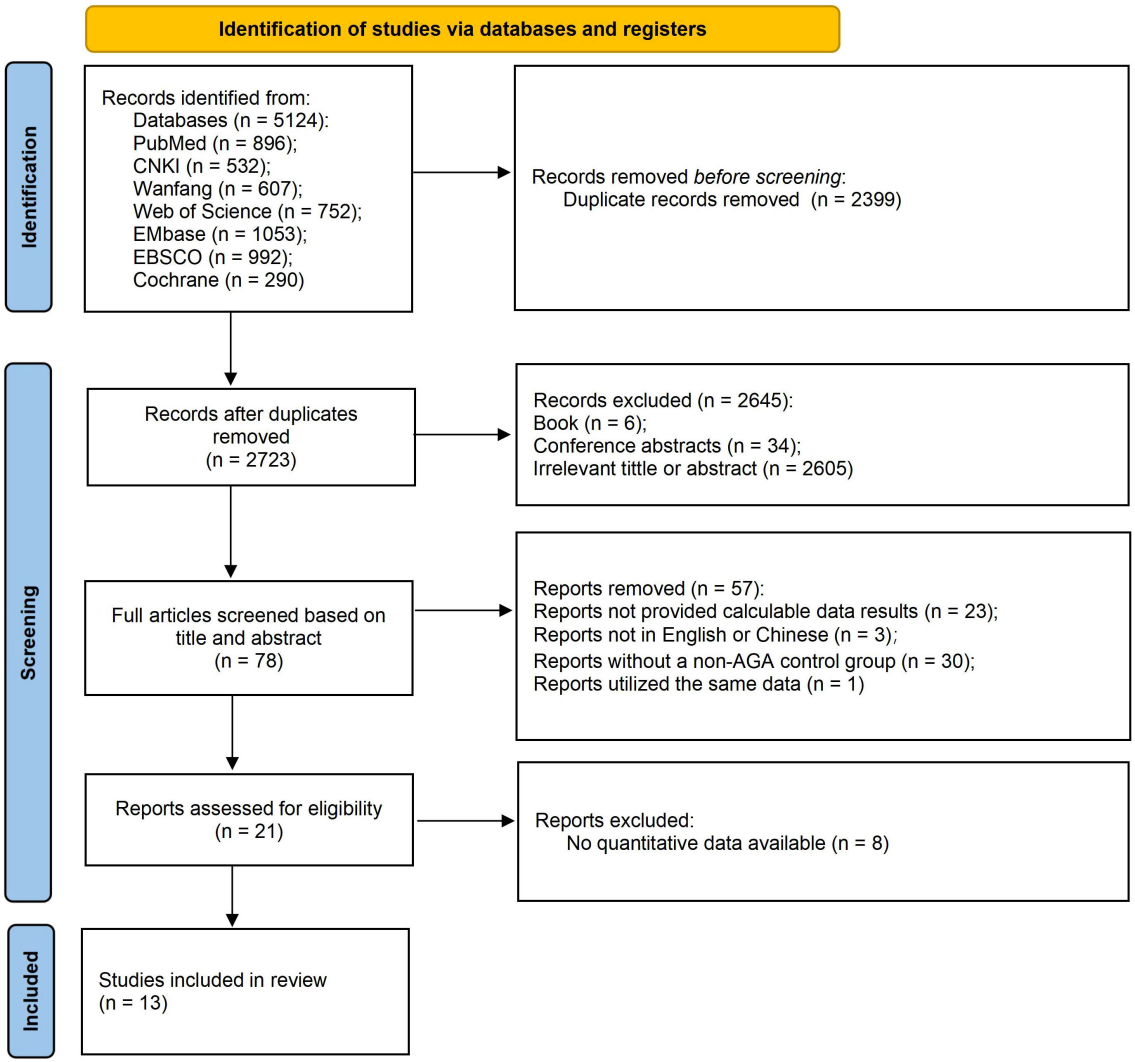
PRISMA flowchart of study selection.

1 case-control study and 12 cross-sectional studies were included. The case-control study received a quality score of 7, while the quality scores of the cross - sectional studies ranged from 4 to 9. All studies were deemed to be of relatively medium quality and thus eligible for inclusion in the meta-analysis. The detailed screening procedure is depicted in **Figure 1**. **Table 1** lists the basic characteristics of the included studies, the observed populations, scale tools, outcome indicators, and quality assessment results.

**Table 1:** The characteristics of all qualified studies in meta-analysis.

Ref. No.	Author	Year	Country	Race	Type	Source of non-AGA group	Population, gender and age of non-AGA group	Source of AGA group	Population, gender and age of AGA group	Psychological status assessment scale	Results of non-AGA group: Mean ± SD	Results of AGA group: Mean ± SD	Quality assessment (AHRQ or NOS)
(20)	Cui	2022	China	Asian	Cross-sectional	University students without AGA	5329; 2034M,3295F; 17-27y	University students with AGA	418; 254M,164F; 17-27y	SAS	33.77 ± 7.51	45.53 ± 3.02	9/11
										SDS	30.67 ± 11.12	39.31 ± 5.77	
(18)	Li	2020	China	Asian	Cross-sectional	Healthy individuals without a history of hair loss	93; 44M, 49F; 32.83 ± 9.74y	AGA patients in the dermatology clinic	201; 108M,93F; 31.05 ± 7.94y	DASS-21	6.49 ± 3.62	9 ± 5.92	6/11
										DASS(D)	2.51 ± 1.67	3.43 ± 2.54	
										DASS(A)	1.82 ± 1.94	2 ± 2.1	
										DASS(S)	2.17 ± 1.46	3.57 ± 2.53	
(24)	Lou et al.	2011	China	Asian	Cross-sectional	Healthy individuals without AGA	434; 327M,107F; 27.86 ± 8.74y	AGA patients in the dermatology clinic	437; 331M, 106F; 29.52 ± 7.76y	SAS	30.81 ± 6.61	32.59 ± 5.82	6/11
										SDS	34.30 ± 9.05	35.24 ± 8.10	
(19)	Peng	2018	China	Asian	Cross-sectional	Healthy individuals without AGA	50; 25M,25F; 35.69 ± 5.47y	AGA patients in the dermatology clinic	50; 40M,10F; 36.69 ± 5.47y	SAS	26.45 ± 3.67	31.56 ± 4.89	6/11
(21)	Si	2020	China	Asian	Cross-sectional	University students without AGA	1418; 491M,927F; 18-30y	University students with AGA	109; 64M,45F; 23.47 ± 3.40y	SAS	43.84 ± 9.17	46.81 ± 11.93	9/11
										SDS	52.28 ± 8.97	52.08 ± 10.40	
(22)	Wang et al.	2013	China	Asian	Cross-sectional	Healthy examinees without hair loss symptoms	46; 46M; 19-67y	AGA patients in the dermatology clinic	45; 45M; 19-60y	SAS	26.500 ± 4.386	28.773 ± 3.483	6/11
							48; 48F; 19-67y		18; 18F; 19-60y	SAS	26.292 ± 3.881	31.579 ± 5.872	
(23)	Wang et al.	2008	China	Asian	Cross-sectional	Patients with superficial fungal infections and no hair loss symptoms	106; 74M,32F; 17-57y	AGA patients in the dermatology clinic	418; 341M,77F; 14-67y	CES-D	10.11 ± 4.25	15.2 ± 8.4	8/11
(29)	Assaf et al.	2013	Canada	Multi-ethnic	Cross-sectional	Individuals from North American normative sample	77; Unknown; Unknown	AGA patient volunteers in the dermatology clinic	35; 9M,26F; 38.4 ± 15.3y	EQ-I			4/11
										Self-Regard	100.9 ± 9.4	96.8 ± 15.9	
										Emotional Self-Awareness	102.1 ± 9.9	99.9 ± 15.1	
										Assertiveness	100.6 ± 11.6	98.3 ± 17.2	
										Independence	100.9 ± 11.0	99.1 ± 16.9	
										Self-Actualization	101.5 ± 9.0	94.4 ± 16.4	
										Empathy	100.2 ± 13.5	95.3 ± 14.6	
										Interpersonal Relationship	100.4 ± 9.9	95.6 ± 15.6	
										Stress Tolerance	99.5 ± 9.1	96.0 ± 15.7	
										Impulse Control	99.9 ± 12.8	99.6 ± 14.2	
										Reality Testing	99.5 ± 10.4	94.9 ± 17.3	
										Problem Solving	100.9 ± 9.9	95.2 ± 15.1	
										Optimism	101.4 ± 8.7	95.9 ± 17.0	
										Happiness	101.2 ± 8.4	95.3 ± 16.8	
(28)	Cash TF et al.	1993	America	Multi-ethnic	Cross-sectional	Patients without AGA, with non-visible, non-STI skin conditions	56; 56F; Average 37.2y	AGA patient volunteers in the dermatology clinic	60; 60M; Average 31.3y	IES			6/11
										Stress intrusion	2.64 ± 3.79	7.65 ± 4.70	
										Stress avoidance	3.41 ± 4.31	8.75 ± 5.42	
										TSBI			
										Self-esteem	59.3 ± 9.1	58.8 ± 9.1	
										SCS			
										Social anxiety	7.79 ± 4.16	8.47 ± 4.27	
										Public self-consciousness	14.84 ± 4.28	15.58 ± 4.44	
										multiple-item indices			
										Life satisfaction	23.4 ± 6.8	22.7 ± 5.3	
										Psychosocial well-being	4.62 ± 0.92	4.52 ± 0.66	
						Patients without AGA, with non-visible, non-STI skin conditions	56; 56F; Average 37.2y	AGA patient volunteers in the dermatology clinic	96; 96F; Average 36.6y	IES			
										Stress intrusion	2.64 ± 3.79	10.55 ± 5.75	
										Stress avoidance	3.41 ± 4.31	10.82 ± 5.14	
										TSBI			
										Self-esteem	59.3 ± 9.1	54.5 ± 11.6	
										SCS			
										Social anxiety	7.79 ± 4.16	10.08 ± 4.64	
										Public self-consciousness	14.84 ± 4.28	15.56 ± 4.26	
										multiple-item indices			
										Life satisfaction	23.4 ± 6.8	20.0 ± 6.7	
										Psychosocial well-being	4.62 ± 0.92	3.91 ± 0.92	
(25)	He et al.	2022	China	Asian	Cross-sectional	University students without AGA	9178; 4761M,4417F; 18.19 ± 0.69y	University students with AGA	49; 38M,11F; 18.29 ± 0.71yy	GAD-2	0.97 ± 1.23	0.76 ± 1.01	7/11
										PHQ-2	0.90 ± 1.26	0.80 ± 1.08	
(27)	Lotfy et al.	2020	Egypt	Egyptian	Cross-sectional	Individuals without AGA confirmed by doctors	100; Unknown; Unknown	Individuals with AGA confirmed by doctors	400; Unknown; Unknown	WHOQOL-BREF			4/11
										Psychological	13.4 ± 3.6	13.3 ± 3.7	
(12)	Noha et al.	2023	Egypt	Egyptian	Case-control	Healthy volunteers without AGA	41; 41F; 26.44 ± 7.38y	AGA patient volunteers in the dermatology clinic	46; 46F; 29.85 ± 9.73y	PSS	21.27 ± 6.44	25.17 ± 5.96	7/9
										BAI	28.88 ± 9.18	28.83 ± 8.09	
										BDI	16.73 ± 6.29	20.39 ± 8.02	
(26)	Wang et al.	2018	China	Asian	Cross-sectional	University student volunteers without AGA	406; Comparable to the AGA group	University students with AGA	355; 340M,15F; 17-40y	SCL-90-R			8/11
										Somatization	1.45 ± 0.47	1.78 ± 0.53	
										Obsessive-compulsive	1.89 ± 0.44	1.74 ± 0.51	
										Interpersonal sensitivity	1.40 ± 0.43	1.53 ± 0.51	
										Depression	1.25 ± 0.44	1.57 ± 0.50	
										Anxiety	1.48 ± 0.37	1.49 ± 0.49	
										Hostility	1.48 ± 0.38	1.49 ± 0.56	
										Phobic anxiety	1.16 ± 0.37	1.43 ± 0.36	
										Paranoid ideation	1.40 ± 0.45	1.44 ± 0.45	
										Psychoticism	1.24 ± 0.38	1.48 ± 0.44	
										Global severity index	1.33 ± 0.40	1.50 ± 0.46	

SD, standard deviation; M, male; F, female; y, years.

### 3.2 Association between AGA and symptoms of anxiety

**Figure 2:**
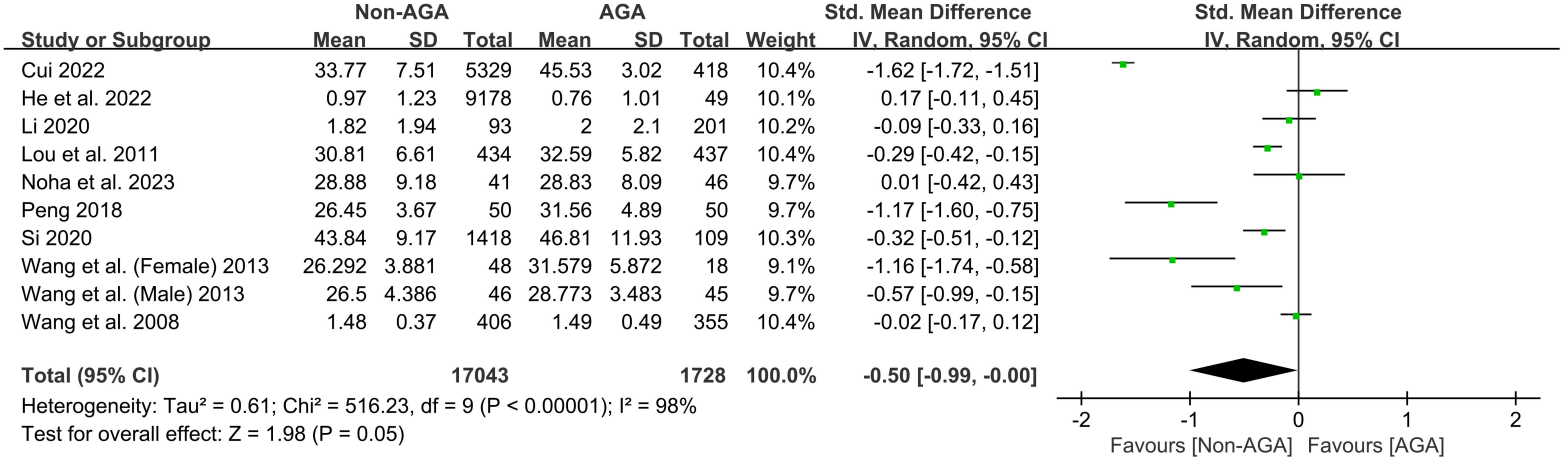
Forest plot of the meta-analysis on the relationship between AGA and symptoms of generalized anxiety.

In the analysis of symptoms of generalized anxiety, a total of 10 studies were included from 9 articles (12, 13, 19–23, 25, 26), involving 17,043 participants in the non-AGA group and 1,728 participants in the AGA group. We employed a random effects model to synthesize the findings from multiple independent studies. The overall effect showed an SMD of -0.50 with a 95% CI of [-0.99, -0.00], indicating that patients with AGA have significantly higher scores on anxiety symptoms compared to those without AGA (**Figure 2**).

**Figure 3:**
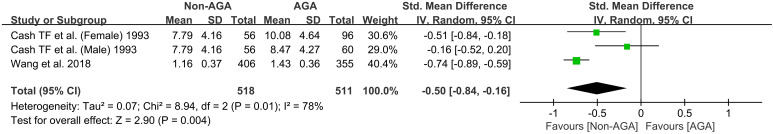
Forest plot of the meta-analysis on the relationship between AGA and symptoms of social anxiety.

As for symptoms of social anxiety, a total of 3 studies in 2 articles (26, 28) were included in the analysis, involving 518 non-AGA individuals and 511 AGA patients. A random effects model was utilized in this study to integrate the results. The pooled SMD was -0.50, with a 95% CI of [-0.84, -0.16], which similarly indicates that patients with AGA have significantly higher scores on symptoms of social anxiety compared to non-AGA patients (**Figure 3**).

### 3.3 Association between AGA and symptoms of depression

**Figure 4:**
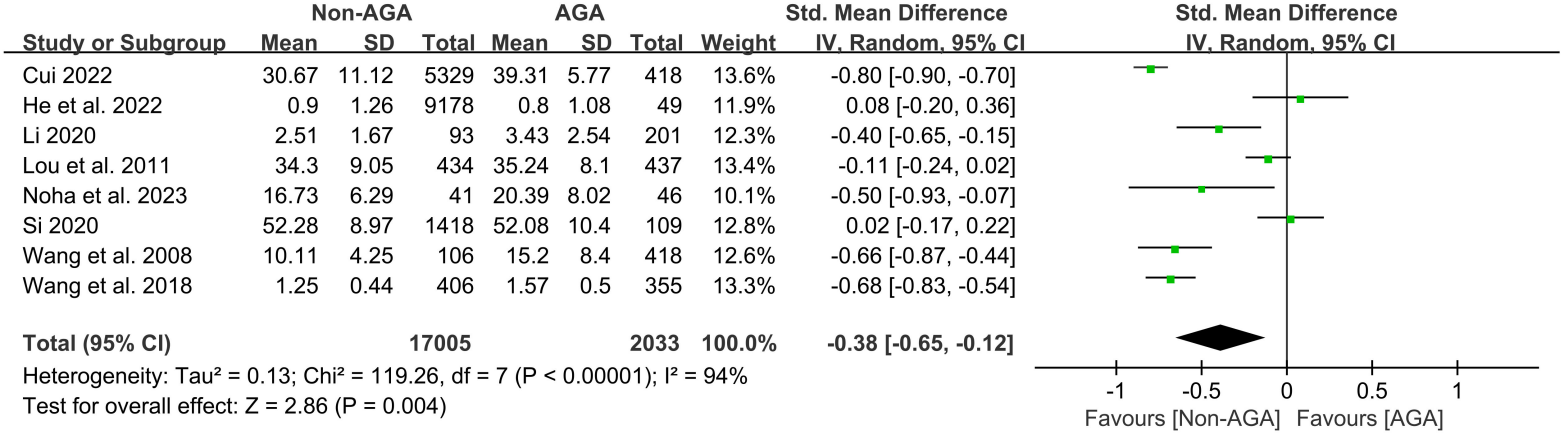
Forest plot of the meta-analysis on the relationship between AGA and symptoms of depression.

8 studies (12, 13, 19, 21, 22, 24–26) were analyzed, including 17,005 non-AGA and 2,033 AGA participants. The results from multiple independent studies were integrated using a random effects model. The overall effect had an SMD of -0.38, with a 95% CI of [-0.65, -0.12], indicating that patients with AGA had significantly higher scores on depression symptoms compared to those without AGA (**Figure 4**).

### 3.4 Association between AGA and perceived stress

**Figure 5:**
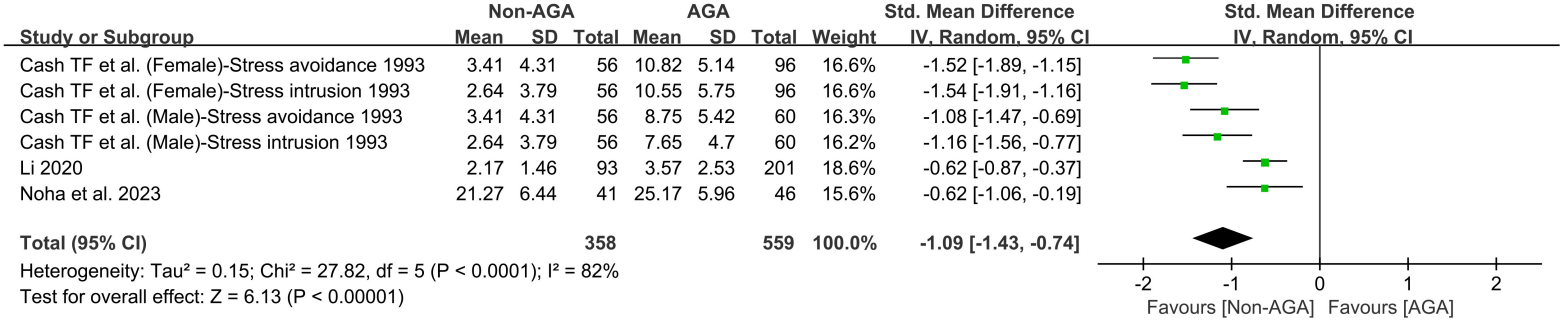
Forest plot of the meta-analysis on the relationship between AGA and perceived stress.

This meta-analysis included 6 studies (12, 19, 28), with a total of 358 participants in the non-AGA group and 559 in the AGA group. A random effects model was used to pool the results. The pooled effect size, represented by an SMD of -1.09, with a 95% CI ranging from -1.43 to -0.74, suggests that individuals with AGA reported significantly elevated levels of perceived stress in comparison to those without AGA (**Figure 5**).

### 3.5 Association between AGA and general distress

**Figure 6:**
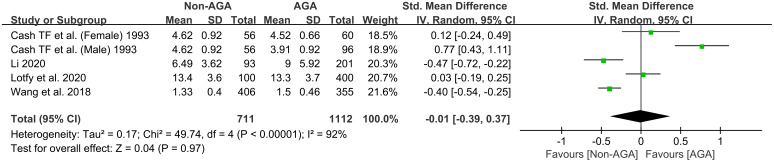
Forest plot of the meta-analysis on the relationship between AGA and general distress.

The meta-analysis incorporated 5 studies (19, 26–28) involving a total of 711 participants from the non-AGA group and 1,112 from the AGA group. Results were pooled using a random effects model. The pooled SMD was -0.01, with a 95% CI ranging from -0.39 to 0.37, indicating that there is no significant difference in the scores of general distress between the AGA group and the non-AGA group (**Figure 6**).

### 3.6 Association between AGA and other conditions of psychological well-being

Cash et al. (28) compared women with AGA, men with AGA, and a female control group, finding that AGA patients, especially women, exhibited more severe body image dissatisfaction, lower self-esteem, and lower life satisfaction. Assaf et al. (29) discovered that AGA patients had lower levels of Self-Actualization, Problem Solving, Optimism, and Happiness in terms of emotional intelligence. Wang et al. (26) conducted a study on 355 Chinese college students and found that the AGA group had higher levels of Somatization, Interpersonal Sensitivity, and Psychoticism, but lower levels of Obsessive-Compulsive behavior.

### 3.7 Publication bias and sensitivity analysis

**Figure 7:**
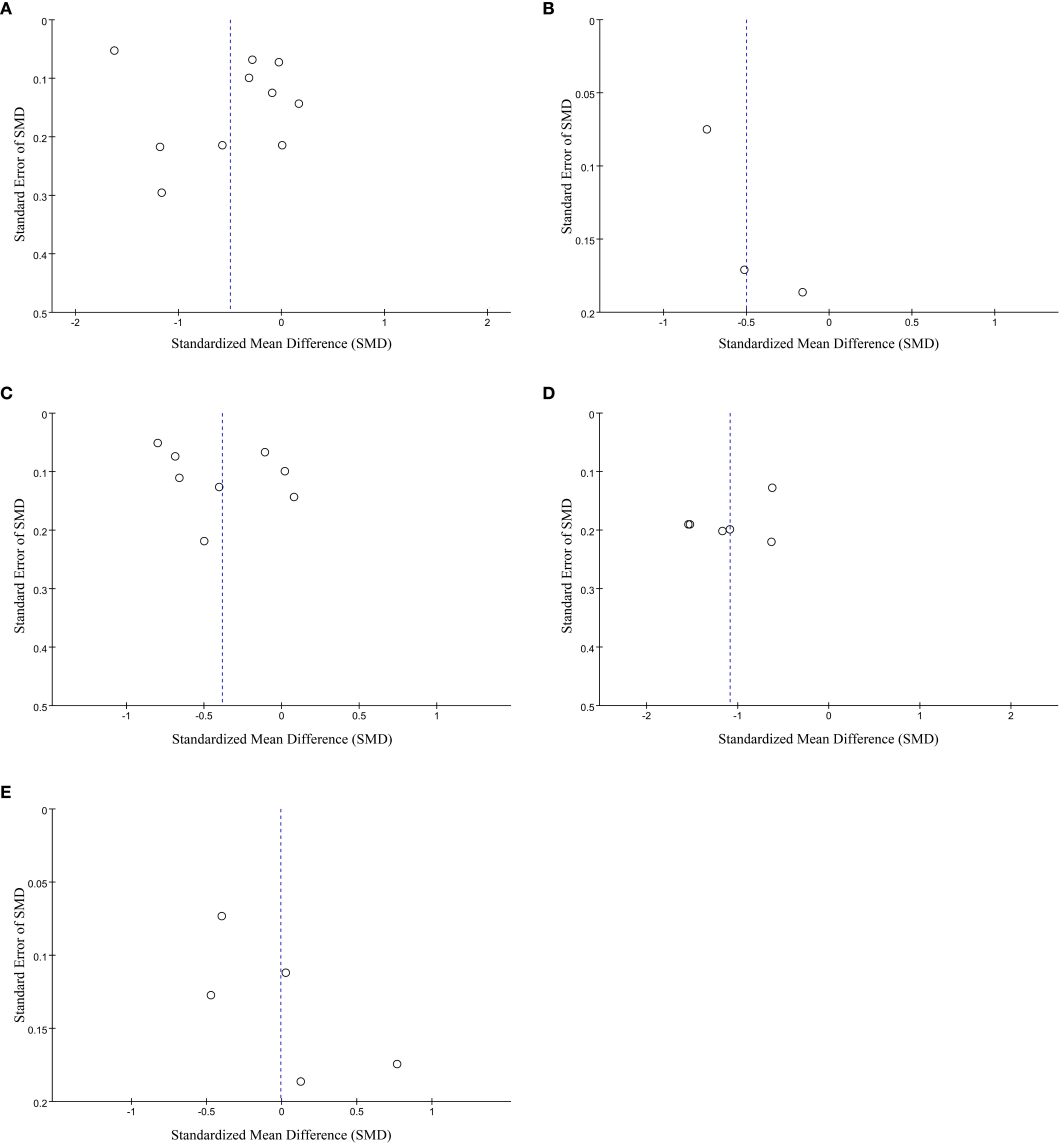
**(A)** Funnel plots of the meta-analysis on the relationship between AGA and symptoms of generalized anxiety. **(B)** Funnel plots of the meta-analysis on the relationship between AGA and symptoms of social anxiety. **(C)** Funnel plots of the meta-analysis on the relationship between AGA and symptoms of depression. **(D)** Funnel plots of the meta-analysis on the relationship between AGA and general distress. **(E)** Funnel plots of the meta-analysis on the relationship between AGA and general distress.

Publication bias analysis was conducted for each dimension, with the funnel plot results illustrated in **Figure 7**. Both Egger’s regression test and Begg’s rank correlation test yielded p > 0.05, indicating minimal publication bias across the studies.

A leave-one-out sensitivity analysis was performed to evaluate the influence of individual studies on the pooled effect and heterogeneity. The exclusion of any single study did not result in significant alterations to the overall effect size estimate or its 95% confidence interval for each meta-analysis, suggesting that the results are not overly reliant on any particular study.

## 4 Discussion

### 4.1 AGA and symptoms of anxiety

We have explored the relationship between AGA and anxiety and found that individuals with AGA tend to have higher levels of anxiety, including both generalized anxiety and social anxiety. Previous research has shown that most men with AGA believe hair loss negatively impacts their social life, and that the more severe the hair loss, the higher the level of anxiety experienced by the patients (30, 31). And it is worth noting that previous studies have identified moderate to severe anxiety as a risk factor for AGA (32). This suggests a possible bidirectional relationship between anxiety and AGA, where anxiety can trigger hair loss, and in turn, hair loss can exacerbate anxiety.

### 4.2 AGA and symptoms of depression

Depression, as a complex and potentially severe psychological well-being state, warrants our attention regarding its association with AGA. The results of this study indicate that the level of depression symptoms in the AGA group is significantly higher. This is similar to the findings of previous studies. For instance, a study involving medical students in India found that the more severe the hair loss, the higher the level of depression (33). A study by Liu et al. in China found that the level of depression in the androgenic alopecia group was significantly higher than that in the non-AGA control group (34).

### 4.3 AGA and stress

Our study reveals that AGA patients have significantly higher stress levels than non-AGA controls. The link between stress and AGA has emerged as a key research focus in the medical field, with multiple studies identifying stress as a significant risk factor for AGA (32, 35).

### 4.4 AGA and general distress

General distress encompasses a range of comprehensive evaluation indicators designed to assess an individual’s overall psychological distress without necessitating a specific psychiatric diagnosis. The meta-analysis results on general distress reveal that, unlike the findings from meta-analyses focusing on specific dimensions, there is no significant difference in overall psychological well-being between the AGA and non-AGA populations. Similarly, Lin et al.’s study also found no significant difference in general distress between these two groups (11). This lack of difference may be attributed to several factors: (a)Limitations of the study sample or methods: The sample size or the specific methodologies employed in the studies may not have been sufficient to detect a significant difference; (b)Indirect impact of AGA on general distress: The influence of AGA on general distress may be mediated by other factors, such as social support or personal coping strategies; (c)Complexity of the concept of general distress: “General distress” is a complex, multidimensional concept that may be challenging to measure accurately with a single indicator.

### 4.5 AGA and other psychological well-being

In this study, AGA patients were found to have lower levels of self-esteem, life satisfaction, and emotional intelligence dimensions such as self-actualization, problem-solving, optimism, and happiness. They also exhibited higher levels of obsessive-compulsive behavior, body image dissatisfaction, somatization, interpersonal sensitivity, and psychoticism. However, due to the limited number of studies focusing on single symptoms, a meta-analysis could not be conducted across these dimensions.

### 4.6 Potential mechanisms underlying the relationship between AGA and psychological well-being

AGA is a multifactorial condition that not only affects hair growth but also has significant psychosocial and biological implications. The potential psychosocial and biological mechanisms underlying the association between AGA and psychological well-being are complex and interconnected.

#### 4.6.1 Psychosocial mechanisms

- Self-image and social anxiety: The visible changes in appearance caused by AGA can create a significant cognitive dissonance in how patients perceive themselves. This can lead to heightened concerns about appearance and the potential stigma associated with hair loss. As a result, patients may become overly preoccupied with negative social evaluations, which in turn can trigger social anxiety (4, 36). This heightened self-awareness and fear of negative judgment can significantly impact an individual’s social interactions and overall mental health;
- The influence of disease cognition: AGA is a condition that can profoundly affect a patient’s sense of control over their health. The lack of understanding of the disease’s pathology and the uncertainty surrounding its progression can lead to feelings of helplessness. Patients who feel they have little control over their condition are more likely to experience higher levels of psychological distress, including increased anxiety and depression (37, 38);
- The role of stress and quality of life: People suffering from hair loss often bear a heavier burden of stress (12, 19), which is further exacerbated by concerns about their hair loss itself (39). This stress not only deteriorates their mental health (40, 41), but also has a significant negative impact on their quality of life. Research has shown a significant positive correlation between quality of life scores and depression symptom scores (24), indicating that a decline in quality of life may worsen depressive symptoms. Moreover, stress, as a state of homeostatic imbalance, often coexists with psychological disorders such as anxiety and depression (40–43), suggesting a potential comorbidity between AGA and adverse psychological states.

#### 4.6.2 Biological mechanisms

- Activation of the hypothalamic-pituitary-adrenal (HPA) axis: Chronic stress activates the HPA axis, leading to persistently elevated cortisol levels that increase the sensitivity of hair follicles to dihydrotestosterone (DHT) and indirectly worsen androgenic damage through neurosteroid synthesis (44–46);
- Inflammatory response: Stress induces the release of pro-inflammatory factors, inhibits the proliferation of hair follicle stem cells, disrupts the hair follicle cycle, and suppresses regenerative signaling in the Wnt pathway (47, 48);
- Oxidative stress: The accumulation of reactive oxygen species (ROS) disrupts the antioxidant defenses of hair follicles, damages the DNA of dermal papilla cells, inhibits the expression of key follicular proteins, and accelerates miniaturization (45, 49);
- Reduction in growth supply: Stress releases epinephrine and norepinephrine, causing vasoconstriction that reduces blood supply to hair follicles. It also alters neurotrophic factor levels, disrupting follicle function (12, 47, 50, 51);
- Genetic amplification effect: Individuals with androgen receptor (AR) gene variants are more sensitive to stress-induced androgen signals, and epigenetic regulation further amplifies genetic risk (45, 52).

#### 4.6.3 Impact of treatment drugs

Existing research indicates that AGA treatment drugs (such as finasteride) themselves may induce psychological side effects such as anxiety, depression, and even suicidal tendencies (53, 54). The confounding effect of the treatment drug should be taken seriously. The potential for treatment-induced psychological side effects underscores the importance of careful monitoring and management of patients undergoing AGA treatment.

In fact, the reasons for the correlation between AGA and a range of mental health issues are complex and multifaceted. This complexity stems not only from the inherent complexity of psychological well-being but also from the influence of sociocultural contexts and personal cognition. For example, in a study by Lin et al. (11), an evaluation of medical staff in a Chinese hospital found that AGA patients exhibited more severe somatization and higher hostility, although they did not have higher levels of interpersonal sensitivity, psychoticism, or lower obsessive-compulsive behavior. And given the variability in environments and self-awareness, AGA patients in specific populations—such as those in certain professions, individuals with high educational levels, unmarried individuals, younger patients, individuals identifying as sexual minority, and those seeking treatment—are particularly prone to psychological well-being issues (4, 26, 55–58). Moreover, it is important to note that some of these symptoms often co-occur and interact, leading to a more complex state of overall psychological well-being for the patients (41, 59). Therefore, research into the mechanisms underlying the relationship between AGA and psychological well-being should be further advanced, and the psychological well-being of AGA patients’ needs to be assessed from multiple dimensions, especially in certain specific populations.

Overall, the relationship between AGA and psychological well-being is multidimensional, complex, and interactive. Firstly, there is a significant association between AGA and various psychological well-being issues, with the mechanisms of influence being multifaceted and intricate. Secondly, this relationship is influenced by the social environment and personal cognition. Last but not least, AGA and psychological well-being are bidirectionally related, as poor psychological well-being can increase the risk of AGA, and hair loss can lead to adverse psychological well-being states in patients.

Future research and clinical practice should focus on the psychological health of AGA patients. First, psychological assessment should be incorporated into the routine clinical process. Standardized psychological scales should be used to comprehensively evaluate patients’ anxiety, depression, stress, and other psychological states, so as to detect and intervene potential problems in time. Second, multidisciplinary collaboration should be strengthened. Dermatologists should work together with psychologists to provide comprehensive treatment plans for patients. For example, patients with significant anxiety and depression symptoms should be referred to psychologists for cognitive-behavioral therapy or pharmacotherapy.

In addition, future research should focus on long-term follow-up of AGA and psychological states. Long-term tracking studies should be conducted to further explore the long-term impact of psychological interventions on the progression of AGA. At the same time, the mechanisms of interaction between AGA and psychological states should be further investigated, including genetic variations and neuroendocrine pathways. Targeted studies should be conducted on specific groups, such as college students and office workers, to analyze the relationship between psychological stress and AGA and explore effective intervention measures. Through these measures, the psychological health of AGA patients can be better improved in the future, and their quality of life can be enhanced.

### 4.7 Strengths and limitations

This systematic review and meta-analysis have several strengths: (a) It is the first systematic review and meta-analysis to examine the relationship between AGA and multiple dimensions of psychological well-being, strictly following PRISMA guidelines; (b) Unlike prior studies that only targeted individuals with AGA, this one systematically compared AGA and non-AGA groups, with both a qualitative review and quantitative analyses; (c) A comprehensive search was conducted across seven databases, including Chinese-language studies, which provided support from Chinese data.

However, this meta-analysis also has some limitations: (a) The studies were restricted to Chinese and English, and the majority of participants were male, resulting in a gender imbalance; (b) Most studies included used a cross-sectional design, with causality requiring further confirmation; (c) The limited number of eligible studies made it challenging to conduct subgroup analyses to explore the sources of heterogeneity and the impact of potential confounding factors; (d) The studies included in this research were conducted across multiple regions, which may introduce potential influences on the results due to cultural differences and self-reporting biases.

## 5 Conclusion

This systematic review and meta-analysis comprehensively evaluated the association between AGA and psychological well-being across multiple dimensions. The findings indicate that AGA is significantly associated with heightened levels of symptoms of generalized and social anxiety, symptoms of depression, perceived stress, body image dissatisfaction, somatization, interpersonal sensitivity, and psychoticism. Additionally, AGA is linked to diminished self-esteem, life satisfaction, emotional intelligence, self-actualization, problem-solving ability, optimism, and happiness. In the future, it is crucial to prioritize patient psychological well-being through assessments and timely interventions. Meanwhile, further research should be conducted to provide precise clinical guidance.

## Data Availability

The original contributions presented in the study are included in the article/**Supplementary Material**. Further inquiries can be directed to the corresponding author.

## References

[1] Su Q Zhou C He C Jiao Q Wang Z Jia Y . Research progress in composition, classification and influencing factors of hair. Asian J Beauty Cosmetol. (2023) 21:503–16. doi: 10.20402/ajbc.2023.0001

[2] Wu X Xu P Zhang Y Zhang Z . Dinguishing human characteristics based on hair metabolomics and proteomics: A review. Chin J Biotech. (2022) 38:3638–47. doi: 10.13345/j.cjb.220526, PMID: 36305399

[3] Frith H Jankowski GS . Psychosocial impact of androgenetic alopecia on men: A systematic review and meta-analysis. Psychol Health Med. (2024) 29:822–42. doi: 10.1080/13548506.2023.2242049, PMID: 37605428

[4] Aukerman EL Jafferany M . The psychological consequences of androgenetic alopecia: A systematic review. J Cosmet Dermatol. (2023) 22:89–95. doi: 10.1111/jocd.14983, PMID: 35403805 PMC10084176

[5] Blume-Peytavi U Blumeyer A Tosti A Finner A Marmol V Trakatelli M . S1 guideline for diagnostic evaluation in androgenetic alopecia in men, women and adolescents. Br J Dermatol. (2011) 164:5–15. doi: 10.1111/j.1365-2133.2010.10011.x, PMID: 20795997

[6] Oiwoh SO Akinboro AO Olasode OA Onayemi EO . Androgenetic alopecia: prevalence and clinical characteristics in a south-west Nigerian population. Nigerian J Med. (2021) 30:507–13. doi: 10.4103/NJM.NJM_102_21

[7] Li G Ding J . Research progress on the pathogenesis of androgenetic alopecia and the western medicine dermal treatment. China Med Her. (2017) 14:43–6+70.

[8] Oiwoh SO Enitan AO Adegbosin OT Akinboro AO Onayemi EO . Androgenetic alopecia: A review. Niger Postgrad Med J. (2024) 31:85–92. doi: 10.4103/npmj.npmj_47_24, PMID: 38826011

[9] Huang C-H Fu Y Chi C-C . Health-related quality of life, depression, and self-esteem in patients with androgenetic alopecia a systematic review and meta-analysis. JAMA Dermatol. (2021) 157:963–70. doi: 10.1001/jamadermatol.2021.2196, PMID: 34232264 PMC8264758

[10] Almashali MA Alotaibi MA Alkhanani AH Al Dera NM Alwadany MM Almousa AS . The psychosocial burden of androgenetic alopecia in Saudi Arabia: A cross-sectional study. J Family Med Prim Care. (2023) 12:3374–9. doi: 10.4103/jfmpc.jfmpc_1151_23, PMID: 38361837 PMC10866284

[11] Lin X Wei Y Lin C Liu C . Investigation and analysis of the current situation of alopecia and its related factors among medical staff in jieyang. J Shantou Univ Med Coll. (2024) 37:58–62. doi: 10.13401/j.cnki.jsumc.2024.01.013

[12] Mohamed NE Soltan MR Galal SA El Sayed HS Hassan HM Khatery BH . Female pattern hair loss and negative psychological impact: possible role of brain-derived neurotrophic factor (Bdnf). Dermatol Pract conceptual. (2023) 13. doi: 10.5826/dpc.1303a139, PMID: 37557155 PMC10412050

[13] He F Shen M Zhao Z Liu Y Zhang S Tang Y . Epidemiology and disease burden of androgenetic alopecia in college freshmen in China: A population-based study. PloS One. (2022) 17:e0263912. doi: 10.1371/journal.pone.0263912, PMID: 35171966 PMC8849549

[14] Sinikumpu SP Jokelainen J Auvinen J Timonen M Huilaja L . Association between psychosocial distress, sexual disorders, self-esteem and quality of life with male androgenetic alopecia: A population-based study with men at age 46. BMJ Open. (2021) 11. doi: 10.1136/bmjopen-2021-049855, PMID: 39192534 PMC8719200

[15] Zeng J He J Chen M Li J . Association between mean platelet volume and obstructive sleep apnea-hypopnea syndrome: A systemic review and meta-analysis. PloS One. (2024) 19:e0297815. doi: 10.1371/journal.pone.0297815, PMID: 38363791 PMC10871486

[16] Linardon J Cuijpers P Carlbring P Messer M Fuller-Tyszkiewicz M . The efficacy of app-supported smartphone interventions for mental health problems: A meta-analysis of randomized controlled trials. World Psychiatry. (2019) 18:325–36. doi: 10.1002/wps.20673, PMID: 31496095 PMC6732686

[17] Higgins JPT TJ Chandler J Cumpston M Li T Page MJ Welch VA eds. Cochrane Handbook for Systematic Reviews of Interventions Version 6.5. In: Cochrane.

[18] Wan X Wang W Liu J Tong T . Estimating the sample mean and standard deviation from the sample size, median, range and/or interquartile range. BMC Med Res Methodol. (2014) 14:135. doi: 10.1186/1471-2288-14-135, PMID: 25524443 PMC4383202

[19] Li Q . A Correlation Study of Negative Emotions and Chinese Medicine Syndrome of Androgenic Alopecia. China: Guangzhou University of Chinese Medicine (2020).

[20] Peng D . Research on the relationship between various alopecia diseases, sleep quality and emotional factors. World Latest Med Inf. (2018) 18:164+8. doi: 10.19613/j.cnki.1671-3141.2018.58.080

[21] Cui W . Investigation and Analysis of Androgenetic Alopecia and Related Factors among College Students in Jiamusi University. China: Jiamusi University (2022).

[22] Si Y . An Investigation on the Prevalence of Androgenetic Alopecia and Its Related Factors in Students of Ningxia Medical University. China: Ningxia Mdedical University (2020).

[23] Wang D Xu Q Lei X Wu X Wu J . Correlation between the three kinds of common hair loss diseases, sleep quality and emotional factors. J Pract Dermatol. (2013) 6:339–42.

[24] Wang L Fan W Cao L Xu W Liu L Li Z . The life quality of patients with alopecia. J Clin Dermatol. (2008) 37:417–9. doi: 10.3969/j.issn.1000-4963.2008.07.002

[25] Lou W Yang Q . A preliminary study on the psychological and psychiatric situation and quality of life on the patients with alopecia. China J Leprosy Skin Dis. (2011) 27:219–20. doi: 10.3969/j.issn.1009-1157.2011.03.041

[26] Wang X Xiong C Zhang L Yang B Wei R Cui L . Psychological assessment in 355 chinese college students with androgenetic alopecia. Med (United States). (2018) 97. doi: 10.1097/MD.0000000000011315, PMID: 30075498 PMC6081179

[27] Elsaie LT Elshahid AR Hasan HM Soultan FAZM Jafferany M Elsaie ML . Cross sectional quality of life assessment in patients with androgenetic alopecia. Dermatologic Ther. (2020) 33:e13799. doi: 10.1111/dth.13799, PMID: 32520416

[28] Cash TF Price VH Savin RC . Psychological effects of androgenetic alopecia on women: comparisons with balding men and with female control subjects. J Am Acad Dermatol. (1993) 29:568–75. doi: 10.1016/0190-9622(93)70223-g, PMID: 8408792

[29] Monselise A Bar-On R Chan L Leibushor N McElwee K Shapiro J . Examining the relationship between alopecia areata, androgenetic alopecia, and emotional intelligence. J cutaneous Med Surg. (2013) 17:46–51. doi: 10.2310/7750.2012.12003, PMID: 23364150

[30] Cash TF . The psychological effects of androgenetic alopecia in men. J Am Acad Dermatol. (1992) 26:926–31. doi: 10.1016/0190-9622(92)70134-2, PMID: 1607410

[31] Hwang HW Ryou S Hyeong Jeong J Lee JW Lee KJ Lee SB . The quality of life and psychosocial impact on female pattern hair loss. Ann Dermatol. (2024) 36:44–52. doi: 10.5021/ad.23.082, PMID: 38325433 PMC10861302

[32] Feng J Zhang Z Xie J Song J . Investigation of influencing factors of androgenetic alopecia in wuhan city. Med J Wuhan Univ. (2023) 44:65–9. doi: 10.14188/j.1671-8852.2022.0520

[33] Prasanna H Rajashekar TS Kumar KS Athish KK Kiran M Reddy M . The association of depression, loneliness, and internet addiction levels in male bachelor of medicine, bachelor of surgery (Mbbs) students with androgenetic alopecia male pattern baldness in a medical college in kolar, India. Cureus J Med Sci. (2023) 15. doi: 10.7759/cureus.35607, PMID: 37007329 PMC10063234

[34] Liu S Gu H Ji R Shi W Liu F Xie H . The mediation role of sleep on the relationship between drinks behavior and female androgenetic alopecia. PeerJ. (2024) 12:e18647. doi: 10.7717/peerj.18647, PMID: 39655323 PMC11627085

[35] Ye X Song X Liu X Zhou W Wang F Chen H . Analysis of influencing factors of female androgenetic alopecia in young women. Chongqing Med J. (2024) 53:69–72+8.

[36] Creadore A Manjaly P Li SJ Tkachenko E Zhou G Joyce C . Evaluation of stigma toward individuals with alopecia. JAMA Dermatol. (2021) 157:392–8. doi: 10.1001/jamadermatol.2020.5732, PMID: 33688916 PMC7948115

[37] Alotaibi HM Idris RB Alajlan AH Alghufaili AA Barakeh M Alobaid SA . Illness perception, psychological distress, and quality of life in patients with alopecia: A cross-sectional study from riyadh, Saudi Arabia. Arch Dermatol Res. (2025) 317:646. doi: 10.1007/s00403-025-04093-2, PMID: 40153034

[38] Yu NL Tan H Song ZQ Yang XC . Illness perception in patients with androgenetic alopecia and alopecia areata in China. J Psychosom Res. (2016) 86:1–6. doi: 10.1016/j.jpsychores.2016.04.005, PMID: 27302539

[39] Li Y Li J Xie Z Zhou L Liu Y Wang W . The relationship between uncertainty stress and perceived anxiety on hair loss among college students in guangzhou. Chin J Soc Med. (2024) 41:72–6. doi: 10.3969/j.issn.1673-5625.2024.01.018

[40] Hou Y Shang M Yu X Gu Y Li H Lu M . Joint effects of recent stressful life events and adverse childhood experiences on perinatal comorbid anxiety and depression. BMC Pregnancy Childbirth. (2023) 23:41. doi: 10.1186/s12884-023-05375-1, PMID: 36653742 PMC9847044

[41] Davies MR Glen K Mundy J ter Kuile AR Adey BN Armour C . Factors associated with anxiety disorder comorbidity. J Affect Disord. (2023) 323:280–91. doi: 10.1016/j.jad.2022.11.051, PMID: 36442657 PMC10202820

[42] Lu S Wei F Li G . The evolution of the concept of stress and the framework of the stress system. Cell Stress. (2021) 5:76–85. doi: 10.15698/cst2021.06.250, PMID: 34124582 PMC8166217

[43] Weinmann T Wibowo R Forster F Gerlich J Wengenroth L Weinmayr G . Association of chronic stress during studies with depressive symptoms 10 years later. Sci Rep. (2025) 15:2379. doi: 10.1038/s41598-025-85311-9, PMID: 39827282 PMC11742973

[44] Craft TK Devries AC . Vulnerability to stroke: implications of perinatal programming of the hypothalamic-pituitary-adrenal axis. Front Behav Neurosci. (2009) 3:54.2009. doi: 10.3389/neuro.08.054.2009, PMID: 20057937 PMC2802556

[45] Lolli F Pallotti F Rossi A Fortuna MC Caro G Lenzi A . Androgenetic alopecia: A review. Endocrine. (2017) 57:9–17. doi: 10.1007/s12020-017-1280-y, PMID: 28349362

[46] McArdle CJ Arnone AA Heaney CF Raab-Graham KF . A paradoxical switch: the implications of excitatory gabaergic signaling in neurological disorders. Front Psychiatry. (2023) 14:1296527. doi: 10.3389/fpsyt.2023.1296527, PMID: 38268565 PMC10805837

[47] Vishnyakova KS Vetkova LG Jasko MV Aliper AM Buzdin AA Popov KV . Hair growth stimulation by a natural remedy: animal studies. Madridge J Dermatol Res. (2018) 3:37–44. doi: 10.18689/mjdr-1000109

[48] Qi J Garza LA . An overview of alopecias. Cold Spring Harb Perspect Med. (2014) 4. doi: 10.1101/cshperspect.a013615, PMID: 24591533 PMC3935391

[49] Diab HM Soltan MY El-Khazragy N Atef Raafat AM . Assessment of the oxidative stress status in androgenetic alopecia in Egyptian men. QJM: Int J Med. (2021) 114. doi: 10.1093/qjmed/hcab093.006

[50] Huan N Yu Y Wang P Wang C . Research progress regarding the diagnosis and treatment of mental stress-induced myocardial ischemia. Anatol J Cardiol. (2020) 24:126–36. doi: 10.14744/AnatolJCardiol.2020.69447, PMID: 32870175 PMC7585978

[51] Cheng Y Lv L-J Cui Y Han X-M Zhang Y Hu C-X . Psychological stress impact neurotrophic factor levels in patients with androgenetic alopecia and correlated with disease progression. World J Psychiatry. (2024) 14:1437–47. doi: 10.5498/wjp.v14.i10.1437, PMID: 39474391 PMC11514570

[52] Sadasivam IP Sambandam R Kaliyaperumal D Dileep JE . Androgenetic alopecia in men: an update on genetics. Indian J Dermatol. (2024) 69:282. doi: 10.4103/ijd.ijd_729_23, PMID: 39119311 PMC11305502

[53] Trüeb RM Gavazzoni Dias MFR Dutra Rezende H . Suicidality and psychological adverse events in patients treated with finasteride. Skin Appendage Disord. (2021) 7:524–6. doi: 10.1159/000514365, PMID: 34901189 PMC8613631

[54] Lyakhovitsky A Amichai B Galili E Cohen A Kridin K Segal Z . The risk of psychiatric disorders in finasteride users with benign prostatic hyperplasia and androgenetic alopecia: A population-based case-control study. Australas J Dermatol. (2024) 65:621–9. doi: 10.1111/ajd.14359, PMID: 39138902

[55] Adamowicz R Załęcki P Dukiel A Nowicka D . Association between androgenetic alopecia and psychosocial disease burden: A cross-sectional survey among polish men. Dermatol Res Pract. (2022) 2022:1845044. doi: 10.1155/2022/1845044, PMID: 35340914 PMC8947924

[56] Zucchelli F Mathews A Sharratt N Montgomery K Chambers J . The psychosocial impact of alopecia in men: A mixed-methods survey study. Skin Health Dis. (2024) 4:e420. doi: 10.1002/ski2.420, PMID: 39355752 PMC11442044

[57] Tas B Kulacaoglu F Belli H Altuntas M . The tendency towards the development of psychosexual disorders in androgenetic alopecia according to the different stages of hair loss: A cross-sectional study. Bras Dermatol. (2018) 93:185–90. doi: 10.1590/abd1806-4841.20185658, PMID: 29723381 PMC5916388

[58] Moorthy SK Yu L Peng L Shen L Han Y Zhang Z . Quality of life and its association with androgenetic alopecia patients in shanghai: A cross-sectional study. Clinical Cosmetic Investigational Dermatol. (2022) 15:2883–93. doi: 10.2147/ccid.s393633, PMID: 36597520 PMC9805703

[59] McGrath JJ Lim CCW Plana-Ripoll O Holtz Y Agerbo E Momen NC . Comorbidity within mental disorders: A comprehensive analysis based on 145–990 survey respondents from 27 countries. Epidemiol Psychiatr Sci. (2020) 29:e153. doi: 10.1017/s2045796020000633, PMID: 32782057 PMC7443806

