# Supplementary material for: Association between androgenetic alopecia and psychological well-being: a systematic review and meta-analysis
Source: Front Psychiatry. 2025 Nov 26;16:1705957. doi: 10.3389/fpsyt.2025.1705957 (PMC12690262; doi:10.3389/fpsyt.2025.1705957)
Supplement: Supplementary file 1 [file Table1.docx]

Supplementary Material

Search strategies across 7 databases for meta-analysis.

| Search terms for PubMed |
| --- |
| #1 ("androgenetic alopecia" OR "hair loss" OR "pattern baldness") |
| #2 ("mental disorders" OR psychology OR psychological OR emotion OR psyche OR "quality of life" OR anxiety OR depression OR stress OR "self-concept" OR self-esteem OR "risk factors") |
| #3 (questionnaire OR scale OR inventory OR index) |
| #1 AND #2 AND #3 |
| Search terms for EBSCO |
| #1 ("androgenetic alopecia" OR "hair loss" OR "pattern baldness") |
| #2 ("mental disorders" OR psychology OR psychological OR emotion OR psyche OR "quality of life" OR anxiety OR depression OR stress OR "self-concept" OR self-esteem OR "risk factors") |
| #3 (questionnaire OR scale OR inventory OR index) |
| #1 AND #2 AND #3 |
| Search terms for Web of Science |
| #1 ((TS=("androgenetic alopecia")) OR TS=("hair loss")) OR TS=("pattern baldness") |
| #2 (((((((((((TS=("mental disorders")) OR TS=(psychology)) OR TS=(psychological)) OR TS=(emotion)) OR TS=(psyche)) OR TS=("quality of life")) OR TS=(anxiety)) OR TS=(depression)) OR TS=(stress)) OR TS=("self-concept")) OR TS=(self-esteem)) OR TS=("risk factors") |
| #3 (((TS=(questionnaire)) OR TS=(scale)) OR TS=(inventory)) OR TS=(index) |
| #1 AND #2 AND #3 |
| Search terms for Embase |
| #1 'androgenetic alopecia':ti,ab,kw OR 'hair loss':ti,ab,kw OR 'pattern baldness':ti,ab,kw |
| #2 'mental disorders':ti,ab,kw OR psychology:ti,ab,kw OR psychological:ti,ab,kw OR emotion:ti,ab,kw OR psyche:ti,ab,kw OR 'quality of life':ti,ab,kw OR anxiety:ti,ab,kw OR depression:ti,ab,kw OR stress:ti,ab,kw OR 'self-concept':ti,ab,kw OR self-esteem:ti,ab,kw OR 'risk factors':ti,ab,kw |
| #3 questionnaire:ti,ab,kw OR scale:ti,ab,kw OR inventory:ti,ab,kw OR index:ti,ab,kw |
| #1 AND #2 AND #3 |
| Search terms for Cochrane |
| #1 ("androgenetic alopecia"):ti,ab,kw OR ("hair loss"):ti,ab,kw OR ("pattern baldness"):ti,ab,kw (Word variations have been searched) |
| #2 ("mental disorders"):ti,ab,kw OR psychology:ti,ab,kw OR psychological:ti,ab,kw OR emotion:ti,ab,kw OR psyche:ti,ab,kw OR ("quality of life"):ti,ab,kw OR anxiety:ti,ab,kw OR depression:ti,ab,kw OR stress:ti,ab,kw OR ("self-concept"):ti,ab,kw OR self-esteem:ti,ab,kw OR ("risk factors"):ti,ab,kw |
| #3 questionnaire:ti,ab,kw OR scale:ti,ab,kw OR inventory:ti,ab,kw OR index:ti,ab,kw |
| #1 AND #2 AND #3 |
| Search terms for CNKI (in Chinese) |
| #1 (TI=hair loss + androgenetic alopecia + seborrheic alopecia OR KY=hair loss + androgenetic alopecia + seborrheic alopecia OR AB=hair loss + androgenetic alopecia + seborrheic alopecia) |
| #2 (TI=psychology + psychological well-being + emotion + psyche + quality of life OR KY=psychology + psychological well-being + emotion + psyche + quality of life OR AB=psychology + psychological well-being + emotion + psyche + quality of life) |
| #3 (TI=questionnaire + scale + inventory + index OR KY=questionnaire + scale + inventory + index OR AB=questionnaire + scale + inventory + index) |
| #1 AND #2 AND #3 |
| Search terms for Wanfang (in Chinese) |
| #1 (Topic: (("hair loss") or ("androgenetic alopecia") or ("seborrheic alopecia"))) |
| #2 (Topic: (("psychology") or ("psychological well-being") or ("emotion") or ("psyche") or ("quality of life"))) |
| #3 (Topic: (("questionnaire") or ("scale") or ("inventory") or ("index"))) |
| #1 AND #2 AND #3 |
